# Socioeconomic determinants of eating pattern of adolescent students in Mansoura, Egypt

**Published:** 2012-10-01

**Authors:** Abdel-Hady El-Gilany, Ghada Elkhawaga

**Affiliations:** 1 Department of Public Health, College of Medicine, Mansoura University, Egypt

**Keywords:** Dietary habits, meal pattern, fast food, Adolescents, students

## Abstract

**Introduction:**

During the last few decades, Egypt experienced rapid socio-cultural changes that were associated with major changes in the food choices and eating habits, which, progressively, becomes more westernized. The objective of this study was to investigate the meal patterns of secondary school adolescent students in Mansoura, Egypt.

**Methods:**

This is a cross-sectional study conducted on 891 adolescent students. Thirty clusters were selected to cover both general and vocational public schools of both sexes in urban and rural areas. A self-administered questionnaire was used to collect data about sociodemographic features of the students and their families, as well as meal habits of students.

**Results:**

About 46% of students eat three meals per day. About 72%, 93% and 95% of respondents consume breakfast, lunch and dinner on daily bases, respectively. Snacks were eaten daily by 34.1% of students. Eating always with the family was stated by the majority (62.5%) of students and taking home made sandwiches during school time was mentioned by 35.8% of students. On logistic regression socioeconomic status is the only predictor associated with daily intake of breakfast, lunch and dinner; with high likelihood of eating with the family and intake of school meal.

**Conclusion:**

Students practice many faulty meal patterns. School-, family- and community-based interventions are timely needed to promote healthy eating habit in adolescents.

## Introduction

During recent decades, almost all the Arab countries have witnessed dramatic lifestyle changes including meal pattern [1, 2]. Egypt has been experiencing a nutrition transition in the context of abundant dietary energy availability, and moderate fat intakes [3]. Childhood and adolescence are important times for establishing healthy dietary habits [4]. During adolescence, young people are assuming responsibility for their own eating habits, health attitudes and behaviors [5].

The health of children and adolescents is dependent upon food intake to promote optimal physical growth, social and cognitive development [6, 7]. Nutritional problems of adolescents, whether under nutrition or related to chronic diseases, are mainly the result of dietary inadequacies. These may be linked to a number of physiological, socio-economic and psychosocial factors. The last group of factors is probably the most important as the search for identity, the struggle for independence and acceptance, and concern about appearance, tends to have a great impact on lifestyle, eating patterns among adolescents [8].

The development of eating behavior is a complex process influences by social, cultural, biological, ecological, and personal factors [9, 10]. From a developmental perspective, societal influence increases as the child ages and parental influences remains important throughout childhood [11].

There is a strong relationship between obesity and food intake and dietary patterns of adolescents. Scientific evidence is increasing about the dietary factors associated with this relationship, specifically a low meal frequency and skipping breakfast [12]. To prevent diet-related chronic diseases, healthy meal habits should be established in childhood and maintained during adolescence.

Recent decades have witnessed the progressive erosion of the traditional Egyptian diet and the introduction of new foods and eating habits. Sociocultural and economic changes are accelerating this erosion [13]. The availability of specific timely and accurate nutritional data is still a problem in Egypt. To the best of the authors’ knowledge there is very little information about meal patterns and how these vary according to sociodemographic characteristics among adolescents in Egypt. Therefore, the objectives of this study are to describe the meal patterns of secondary school adolescent students in Mansoura, Egypt and to explore their association with sociodemographic factors. This information will help health educators and policy makers to develop proper nutrition- related education programs that promote good eating habits.

## Methods

### Design and sample

This is a cross-sectional study carried out during the period October 1, 2010 - December 1, 2010 in Mansoura, the capital city of Dakahlia governorate, Egypt, located on the river Nile in the northeast of the Delta. Approval of the local directorate of education and school administration was obtained. The study protocol was approved by Research Ethics Committee, College of Medicine; Mansoura University. In Egypt parental consent is only required in case of invasive procedures. The survey was carried out among secondary school students enrolled in general and vocational public schools. All over the country including Mansoura, students enrolled in private school are a minority (about less than 10% of all student population) and mostly belong to families of high social standards with high income to pay for school. Private schools are only available at large cities and not in rural areas or small towns.

The sample size was calculated using Epi-Info, version 6.02. The total number of students registered in the secondary schools of Mansoura district was about 650 000 (according to the directorate of education). The pilot study on 120 students (not included in the full-scale study) indicated that about 21% of students were not eating breakfast in daily basis. With the worst acceptable level 18%, the sample needed for the study was estimated to be at least 707 at 95% confidence level. Secondary schools in both educational zones (eastern and western zones) in Mansoura city as well as the rural sector were included. One general secondary school for girls and 1 for boys were randomly selected from each zone (i.e. 4 general schools in the urban sector) as well as 1 mixed school from the rural sector. Five vocational schools (1 commercial school for boys and 1 for girls; 1 industrial school for boys and 1 for girls and 1 mixed agricultural school) were selected from Mansoura city. This distribution covered all social strata, both sexes, and included both urban and rural sectors of the community. From each selected school, 1 class (cluster) from each grade was randomly selected, i.e. 30 classes in all, 10 from each grade. A total of 993 students were registered in these classes and 891 participated in the study (response rate of 89.7%). The others were either absent (8.2%), refused to complete the questionnaire (1.7%) or excluded from the study due to chronic diseases or disability (0.4%).

The investigators spent about 30 minutes in each class. Students were briefed about the study, encouraged to participate and to express their experiences. It was emphasized that all data collected was strictly confidential and the students gave fully informed verbal consent to participate.

### Data Collection

Students completed a self-administered questionnaire on family and personal background, their meal pattern. Students were requested to specify number of their daily meals, snacks, eating with family and diet at school during a typical week. Periods of religious festivals and social events were excluded. The social score was calculated according to Fahmy and El-Sherbiny [14]. This score encompasses the parental education and occupation, per capita income, family size, crowding index at home and the available household appliances and equipment. The total score was categorized into four levels of social class.

### Data Analysis

Data were analysed using SPSS, version 16. Variables were presented as number and per cent. Chi squared test was used for comparison between groups. Logistic regression analysis using forward Wald method was done to find out the independent predictor of each meal pattern. Odds ratio (OR) and their 95% confidence (CI) were calculated. P=0.05 was considered significant.

## Results

Data of 891 students were analysed. Their age ranged from 14 to 20 with a mean of 15.8 ±1.1 years. In a typical week about 46% of students eat three meals per day. Breakfast was consumed by 71.6% of students on daily basis, while daily intake of lunch and dinner were reported by 92.6% and 94.6% of students, respectively. Snacks were eaten daily by 34.1% of students. Eating always with the family was stated by the majority (62.5%) of students and taking home made sandwiches during school time was mentioned by 35.8% of students (Table 1).

**Table 1:** Overall meal pattern of adolescent students in Mansoura, Egypt

	Number (%)
**Number of meals/day**	
1	23(2.6)
2	321(36.0)
3	406(45.6)
4 and 5	141(15.8)
**Breakfast**	
Never	32(3.6)
1-6 days /week	221(24.8)
Daily	638(71.6)
**Lunch**	
Never	7(0.8)
1-6 days /week	59(6.6)
Daily	825(92.6)
**Dinner**	
Never	7(0.8)
1-6 days /week	41(4.6)
Daily	843(94.6)
**Snacks**	
Never	395(33.1)
1-6 days/week	292(32.8)
Daily	304(34.1)
**Eat with family**	
Never/rarely/sometimes	118(13.2)
Frequently	216(24.2)
Always	557(62.5)
**Diet ate during school times**	
None	131(14.7)
School meal	218(24.5)
Homemade sandwiches	319(35.8)
Buy food	223(25.0)

Table 2 shows that the daily intake of three meals or more was significantly higher among students of general schools, from urban areas, of female sex, of working or highly educated mothers, and high socioeconomic status. Daily intake of breakfast was significantly higher among students of vocational schools, of highly educated mothers, and middle socioeconomic status. Daily intake of lunch was significantly higher among students of general schools, of female sex, of working or highly educated mothers, and high socioeconomic status. Daily intake of dinner was significantly higher among students from urban areas, of female sex, of working or highly educated mothers, and high socioeconomic status. A daily snack was significantly higher among students of male sex. Always eating with the family was significantly higher among students from rural areas, of female sex, of housewives or less educated mothers, and low or very low socioeconomic status. Eating during school time was significantly higher among students of vocational schools, from urban areas, of male sex, of highly educated mothers, and high socioeconomic status.

**Table 2:** Meal pattern of adolescent students according to their sociodemographic features

	Total	3 meals or more per day	Daily breakfast	Daily lunch	Daily dinner	Daily snacks	Always eat with family	Diet at school
**Overall**	891	547(61.4)	638(71.6)	825(92.6)	843(94.6)	304(34.1)	557(62.5)	760(85.3)
**School**								
General	514	331(64.4)	336(65.4)	509(99.0)	489(95.1)	179(34.8)	311(60.5)	414(80.5)
Vocational	377	216(57.3)	302(80.1)	316(83.8)	354(93.9)	125(33.2)	246(65.3)	346(91.8)
Significance		P=0.03	P=0.001	P=0.001	P=0.4	P=0.6	P=0.1	P=0.001
**Residence**								
Rural	384	208(54.2)	282(73.2)	349(90.9)	349(90.9)	130(33.9)	256(66.7)	301(78.4)
Urban	507	339(66.9)	356(70.2)	476(93.9)	494(97.4)	174(34.3)	301(59.4)	459(90.5)
Significance		P=0.001	P=0.3	P=0.1	P=0.001	P=0.9	P=0.03	P=0.001
**Sex**								
Boys	443	255(57.6)	329(74.3)	399(90.1)	411(92.8)	167(37.7)	221(49.9)	398(89.8)
Girls	448	292(65.2)	356(70.2)	426(95.1)	432(96.4)	137(30.6)	336(75.0)	362(80.8)
Significance		P=0.02	P=0.1	P=0.004	P=0.02	P=0.025	P=0.001	P=0.001
**Mother's work**								
Housewife	585	333(56.9)	424(72.5)	523(89.4)	543(92.8)	193(33.0)	358(65.8)	492(84.1)
Working	306	214(69.9)	214(69.9)	302(98.7)	300(98.0)	111(36.3)	172(56.2)	268(87.6)
Significance		P=0.001	P=0.4	P=0.001	P=0.001	P=0.3	P=0.05	P=0.2
**Mother's education**								
Below secondary	479	259(54.1)	345(72.0)	417(87.1)	436(91.0)	152(31.7)	328(68.5)	395(82.5)
Secondary	160	104(65.0)	125(78.1)	157(98.1)	158(98.8)	58(36.2)	109(68.1)	129(80.6)
Above secondary	250	184(73.0)	168(66.7)	251(99.6)	249(98.8)	84(37.3)	120(47.6)	236(93.7)
Significance		P=0.001	P=0.04	P=0.001	P=0.001	P=0.3	P=0.001	P=0.001
**Socioeconomic class**								
High	274	198(72.3)	186(67.9)	273(99.6)	271(98.9)	102(37.2)	121(44.2)	254(92.7)
Middle	157	97(61.8)	129(82.2)	156(99.4)	152(96.8)	54(34.4)	117(74.5)	133(84.7)
Low and very low	460	252(54.8)	323(70.2)	396(86.1)	420(91.3)	148(32.2)	319(69.3)	373(81.1)
Significance		P=0.001	P=0.004	P=0.001	P=0.001	P=0.4	P=0.001	P=0.001

However, the logistic regression analysis revealed that enrollment in vocational education is associated with is associated with high likelihood of eating school meals (OR=4.4). Also students of urban residence are two times more likely to eat school meal than those of rural residence. Girls are less likely to consume daily snacks and eat school meals than boys (OR=0.7 and 0.5; respectively). Maternal education is the only independent significant predictor of daily intake of 3 meals or more (OR=1.6 and 2.3 for secondary and above secondary education, respectively) and daily intake of dinner (OR=7.8 and 8.2 for secondary and above secondary education, respectively). Low socioeconomic status are associated with less probability of daily intake of breakfast (OR=0.6), lunch (OR=0.9), and intake of school meal (OR=0.2). However, it is more likelihood of eating with family (OR=4.6) (Table 3).

**Table 3:** Logistic regression analysis of significant independent socioeconomic predictors of meal pattern among adolescent students

	3 meals or more per day OR(95%CI)	Daily breakfast OR(95%CI)	Daily lunch OR(95%CI)	Daily dinner OR(95%CI)	Daily snacks OR(95%CI)	Always eat with family OR(95%CI)	Diet at school OR(95%CI)
**School**							
General							1(r)
Vocational							4.4(2.7-7.1)***
**Residence**							
Rural							1(r)
Urban							2.0(1.3-3.3)**
**Sex**							
Boys					1(r)		1(r)
Girls					0.7(0.6-0.9)*		0.5(0.3-0.8)**
**Mother's education**							
Below secondary	1(r)			1(r)			
Secondary	1.6(1.1-2.3)*			7.8(1.9-32.5)***			
Above secondary	2.3(1.7-3.2)***			8.2(2.5-26.7)***			
**Socioeconomic class**							
High		1(r)	1(r)			1(r)	1(r)
Middle		1.7(1.1-2.8)*	1.5(0.1-25.0)			4.4(2.8-7.0)***	0.9(0.3-2.2)
Low and very low		0.6(0.4-0.9)*	0.6(0.1-0.7)*			4.6(3.2-6.6)***	0.2(0.1-0.7)*

OR=Odds Ratio CI=Confidence Interval r= reference group

* P≤0.05

** P≤0.01

*** P≤0.001

## Discussion

There are three meals consumed on a daily basis in the Egyptian context, in which lunch is the main family meal. This study showed that meal skipping is common among school adolescent (more than 38% of respondents skip one or more meal daily). Breakfast was the meal most frequently skipped. Previous studies reported that reasons for skipping breakfast include lack of time, early school activities, irregular schedule or a poor appetite first thing in the morning [15–18]. Adolescent, particularly females may use skipping meals as a strategy for weight control [19].

More than 38% of respondents skipped one or more meal per day and 15.8% had more than three meals per day. A recent Egyptian study reported that despite high incidence of skipping breakfast, adolescent female students had more than three meals per day [20]. Up to about 60% were reported among Turkish and Gaza adolescents skipped meal at least once a day [6, 21]. Maternal education is the only independent predictor of eating three meals or more per day. This is in contrary to the results of Gaza study [21]. Breakfast and lunch are the meals most often missed, but social, school, and private lessons activities can cause evening meals to be missed as well [8, 15]. Adolescent regular breakfast consumption significantly predicted young adult breakfast consumption and provide considerable protection from obesity during adolescent and young adulthood [22].

In Egypt, the first meal of the day is small and is made up of a drink taken alone or accompanied by a piece of bread [13]. About 72% of respondents reported daily breakfast consumption. The Survey of Young People in Egypt (SYPE) showed that about 57% of the respondents the habit of taking breakfast daily and 3.5% reported never getting breakfast in the morning [23]. In Gaza 62% of adolescents eat breakfast daily [21]. About 50% and 80% of Turkish and Italian adolescents skip breakfast, respectively [6, 24]. A study of eating behaviour of children in Liverpool showed that adolescents, especially girls regularly skip breakfast. The missed breakfast was replaced by eating convenience food on the way to school [25].

Daily intake of breakfast varies with the socioeconomic status of the family. This is in contrary to the results of SYPE study in Egypt [23]. Daily consumption of lunch and dinner were reported by 92.6% and 94.6% of respondents; respectively. Gaza Adolescents reported that lunch was the meal eaten most often, more than 80% had lunch daily. On the other hand, dinner was eaten less often, with only half of the sample having this meal daily [21].

Meal skipping has been shown to be associated with a higher snacking frequency among adolescents [26]. There is a rapidly growing number of working women at all social levels. Children who return from school at an earlier time than their working parents do not always have a meal upon their arrival [13]. These children tide this period with a sandwich, a soft drink, candy, or any of the rapidly growing arrays of junk foods [27]. In Egypt a food habit that has survived till today is that of nibbling different snack. Nibbling is favoured following the evening meal, and snacks are more often consumed outdoors during the long summer evenings. With television watching, nibbling can continue late into the night. Traditional snack foods comprise a variety of different food items and continue to be enjoyed by the rich and the poor of all ages. Some of them are highly nutritious. Their replacement by sweets, candy and junk foods is much less advantageous to the food system [13].

Most adolescents snack. In this study more than one-third of respondents consume snake on daily basis. Snacks are a major source of energy and nutrients, providing nearly one-third of energy intake for many adolescents [19]. Depending upon their timing and composition, between-meal snacks can contribute in negative or positive ways to the adolescent diet. Poorly timed snacks that are high in calories and low in nutrients (i.e. junk food) may blunt the adolescent's mealtime appetite and replace nutritious foods [8, 15, 28]. In contrast, healthy snacks can help to meet the increased energy and nutrient needs of adolescence [15]. In Turkish adolescents: 48.6% choice fast food as snacks, 37% use cakes and sweets in snacks [6].

The importance of parental influence on a child's nutritional status and the development of food preferences was emphasized. Repeated exposure to certain foods influences a child for that food and can shape behaviour later in life [11]. Despite increasing independence in food choices as a child ages, parental influences remains high through adolescence [29]. Adolescents’ busy lifestyles often conflict with family mealtimes [8, 15]. One study demonstrated that children who eat meals with their family most or all of the times have healthier diets (less fried food and soft drinks and more fruits, vegetables and whole grains) than do those who rarely or never do [30]. In Middle East culture sharing food involves symbolism and meanings. This study revealed that 62.5% of students always eat with their families. Food intake with the family is significantly higher among adolescents of middle (OR=4.4) and low or very low socioeconomic status (OR=4.6). In Turkish adolescents: about 80% eat at table with family [6].

It is widely accepted that eating healthy food improves student's concentration, attendance, cognitive functioning and academic performance [23, 31, 32]. More than 85% of students consume a school diet. Only 24.5% consume school meal; 35.8% eat homemade sandwiches and 25% buy their own food. These findings contradict the SYPE results. Only 3.6% of SYPE respondents get a school meal, mainly among those of rural residence and lowest wealth status. Another 74.6% buy their own food and 48.6% bring home-made sandwiches [23].

This data may be useful for early detection of students at risk of faulty meal pattern and targeting them by appropriate interventions. School-, family- and community-based interventions are timely needed. Regular family and school meals could serve as role models for healthy eating behaviour. There is a strong need for further research to understand the behavioural, psychological and cultural factors contributing to unhealthy meal habits and to develop effective intervention for promoting healthy eating during this critical developmental period. Adolescents should be advised not to skip meals, particularly breakfast, eat regular meals using the food pyramid and eat nutritious snacks.

### Study limitations

This study included only students enrolled in public secondary schools, students in private schools and out of school adolescents were not involved. A second limitation is that the study was done one locality (Mansoura) so its results cannot be generalized to the national level. Despite these limitations this is the first study of meal pattern of adolescents in our locality. It may pave the way for large scale national community-based survey to give the picture of adolescents’ diet and nutrition.

## Conclusion

Students practice many faulty meal patterns. About 46% of students eat three meals per day. About 72% of respondents consume breakfast on daily bases, respectively. Snacks were eaten daily by 34.1% of students. Eating always with the family was stated by 62.5% of students and taking home made sandwiches during school time was mentioned by 35.8% of students. On logistic regression socioeconomic status is the only predictor associated with daily intake of breakfast and is associated with high likelihood of eating with the family and intake of school meal. School-, family- and community-based interventions are timely needed to promote healthy eating habit in adolescents.
